# Adiponectin and gastric fundus: A potential target for gut–brain axis dysfunctions

**DOI:** 10.14814/phy2.70398

**Published:** 2025-06-01

**Authors:** R. Garella, F. Palmieri, F. Chellini, L. Tarchi, V. Ricca, G. Castellini, C. Sassoli, R. Squecco

**Affiliations:** ^1^ Department of Experimental and Clinical Medicine, Section of Physiological Sciences University of Florence Florence Italy; ^2^ Department of Experimental and Clinical Medicine, Section of Anatomy and Histology, Imaging Platform University of Florence Florence Italy; ^3^ Department of Health Sciences, Psychiatry Unit University of Florence Florence Italy

**Keywords:** adiponectin, appetite, eating disorders, gastric smooth muscle, inflammatory bowel disease (IBD), obesity

## Abstract

Adiponectin (ADPN) is a pleiotropic hormone produced by adipose tissue involved in the control of body weight, energy expenditure, and feeding behaviors. Alongside a central effect, ADPN acts on peripheral organs such as the stomach, where it can favor gastric fundus relaxation, reinforcing central satiety signals. Notably, ADPN serum levels are dysregulated in several conditions affecting the gastrointestinal tract. Moreover, altered hormone levels coexist with disorders related to the gut–brain axis malfunctioning, that is, eating disorders and inflammatory bowel diseases. Aiming at considering the effective utility of ADPN in a wide range of clinical conditions, there is an urgent need to identify its targets, clarify its mechanism of action, and downstream effectors. In this view, the present review highlights the advancement in elucidating ADPN effects on gastric fundus, describing its ability to cause morphofunctional alterations of smooth muscle cells, affecting their excitability, contractile machinery, and motor response. This comprehensive overview also provides a critical appraisal on the potential translational applications, including the possibility to consider ADPN as a biomarker for the diagnosis and staging of different clinical conditions. Finally, this review explores the potential employment of ADPN analogues for treating disorders characterized by functional gastric disturbances or altered feeding behaviors.

## 1 INTRODUCTION

In recent years, an increasing number of studies have focused on the key role of adipokines in physiological and pathological situations (Tarchi, Garella, et al., 2024). These pleiotropic hormones, typically produced by white adipose tissue, have been proposed as potential biomarkers for the diagnosis and staging of a variety of clinical conditions (Kishida et al., 2014; Mather & Goldberg, 2014), as well as prognostic biomarkers of treatment response (Kusminski & Scherer, 2009). Adipokine analogues have also been suggested as offering a treatment for conditions characterized by functional gastric disturbances or alterations in feeding behaviors (Di Raimo et al., 2015).

Among the different adipokines, adiponectin (ADPN) is particularly attractive. In fact, ADPN is dysregulated in many disorders, especially those related to the gastrointestinal tract, energy metabolism, weight control, or feeding behaviors. For example, we can list inflammatory bowel disease (IBD) (Karmiris et al., 2005), irritable bowel syndrome (IBS) (Baram et al., 2020), metabolic syndrome, obesity, type 2 diabetes mellitus (T2DM), nonalcoholic fatty liver disease (NAFLD) (Duan et al., 2025), gastroesophageal reflux disease, Barrett's esophagus (Duggan et al., 2013), anorexia nervosa (AN), bulimia nervosa (BN), and binge eating disorder (BED) (Karageorgiou et al., 2020; Monteleone et al., 2003).

Aiming to use ADPN in the early diagnosis, prognosis, or treatment of several diseases, and to clearly state potential translational perspectives, it is necessary to clarify its target organs, which membrane receptors are recruited, and its mechanisms of action, including triggered signaling pathways. For this purpose, the present review synthesizes the current knowledge on the role of ADPN in gastric function and appetite regulation, critically evaluating its potential as a biomarker and gastrointestinal disorders, and advancing preliminary hypothesis for future research on the potential role of ADPN in metabolic or feeding disorders.

## 2 STRUCTURE AND FUNCTIONS OF ADPN

ADPN is a polypeptide of 244 amino acids (30 kDa) mainly secreted by adipocytes, but also by cardiomyocytes, endothelial, and skeletal muscle cells. These observations were validated both in human and in murine tissues (Ding et al., 2012).

In both species, ADPN circulating in serum can be found in different complexes such as trimers or low‐molecular‐weight (LMW), hexamers or medium‐molecular‐weight (MMW), as well as high‐molecular‐weight (HMW) multimers (Schraw et al., 2008; Waki et al., 2003; Whitehead et al., 2006). Trimers and HMW multimers trigger different signaling paths, while their relative amounts are altered in obesity (Engin, 2017). In addition to taking part in glucose and lipid metabolism (Yanai & Yoshida, 2019), and in maintaining energy homeostasis (Lee & Shao, 2014), ADPN exerts a protective role in some common pathologies. In fact, several studies reported an antidiabetic effect in case of insulin resistance (Ziemke & Mantzoros, 2010), anti‐obesogenic effects in metabolic disorders (Uddin et al., 2021), as well as anti‐inflammatory and anti‐atherogenic actions—reducing the expression of proinflammatory cytokines (Ouchi & Walsh, 2007; Orlando et al., 2019). Besides these functions, preliminary evidence suggests an antineoplastic role of ADPN in different cancers, such as breast or gastric neoplasia (Ming et al., 2023; Nehme et al., 2022). Additionally, low ADPN levels, as observed in obese subjects, have been correlated with more rapid tumor growth (Capuozzo et al., 2023; Parida et al., 2019).

However, evaluations of the hormone circulating levels may provide different results based on the species analyzed: in humans for instance, circulating ADPN concentrations are lower than those in mice and are inversely correlated with body mass index (BMI) or insulin resistance. In humans, individual factors such as gender (females generally have higher levels than males) and genetic variations can influence ADPN concentrations (Blüher et al., 2006; Hui et al., 2012; Jasinski‐Bergner et al., 2017). On the other hand, mice typically exhibit higher circulating ADPN levels compared to humans, which is thought to contribute to the enhanced insulin sensitivity observed in rodents (Bullen Jr. et al., 2007).

## 3 ADPN RECEPTORS AND SIGNALING PATHWAYS

The activity of ADPN is mediated by three different receptors: AdipoR1, AdipoR2, and T‐cadherin. All are widely expressed in different tissues within mammals (Yamauchi & Kadowaki, 2008), as well as in both humans and rodents, with similar tissue distributions. In particular, AdipoR1 is mainly expressed in skeletal muscle; AdipoR2 is mainly expressed in the liver; and T‐cadherin is mainly expressed in the spinal cord, aortic walls, and neurons of the cerebral cortex. All of these receptors are expressed in mammalian cardiac tissue (Hug et al., 2004; Yamauchi et al., 2003). Our research group has recently observed the presence of both AdipoR1 and AdipoR2 receptors in the inner circular and external longitudinal muscular layers of murine gastric fundus, with AdipoR2 being the most represented among the two (Garella, Bernacchioni, et al., 2023).

In regards to the possible mechanisms of action, the classic inhibitory pathway of nitric oxide (NO) seems to be involved in ADPN's ability to influence the relaxant mechanical responses of murine gastric fundus samples (Idrizaj et al., 2018). Following binding with its receptors, ADPN recruits the NO/guanylate cyclase pathway to modulate murine gastric smooth muscle cell functional features (Garella, Cassioli, et al., 2023). ADPN was also shown to exert its inhibitory action on gastric smooth muscle by recruiting the 5'‐AMP‐activated protein kinase (AMPK) signaling pathway (Idrizaj, Garella, Nistri, et al., 2020; Idrizaj, Garella, Squecco, & Baccari, 2020), as evaluated by the electrophysiological and biophysical properties of murine samples. Furthermore, a ceramidase enzymatic activity associated with AdipoR has also been previously described in murine hepatic cells (Holland et al., 2011). In fact, a decrease in cellular ceramide has been observed following AMPK activation (Chaurasia & Summers, 2015). This is due to the hydrolytic activity of ceramidases, converting ceramides into sphingosine which, in turn, can be phosphorylated to sphingosine‐1‐phosphate (S1P) by sphingosine kinase (SK). In subsequent studies, crystal structures of human AdipoRs were demonstrated to possess an intrinsic basal ceramidase activity enhanced by ADPN (Vasiliauskaité‐Brooks et al., 2017).

Both ceramides and S1P have been found to be dysregulated in obese subjects (Brown et al., 2024), supporting the hypothesis that ADPN contributes to energy homeostasis control. Recently, our research group demonstrated the recruitment of SK/S1P signaling upon ADPN‐AdipoR binding in the murine gastric fundus muscular tissue. Among the two isoforms of SK (SK1 and SK2) expressed in this smooth muscle tissue, SK2 seems to be the one mostly involved in ADPN activity, since its pharmacological inhibition determines major alterations of ADPN effect (Garella, Bernacchioni, et al., 2023).

Studies in murine models confirmed that AdipoR1 and AdipoR2 interact with globular and full‐length ADPN, mediating increased AMPK/PPARα ligand activities, glucose uptake and fatty‐acid oxidation (Yamauchi et al., 2007). Both the receptors are associated with an adaptive protein, namely “adaptor protein, phosphotyrosine interacting with PH domain and leucine zipper 1” (APPL1), which has been shown to be involved in different pathways. AdipoR1‐APPL1 interaction is linked to several molecular processes, such as the promotion of glucose transporter (Glut‐4) translocation (with the consequent reduction of glycemia) or AMPK‐mediated pathways, vasodilation, cytoprotection, and β‐oxidation. Moreover, AdipoR1‐APPL1 interaction shows a crosstalk with the analogue pathway from insulin receptors, leading to several effects on metabolism, such as an increase in protein and glycogen synthesis, gluconeogenesis, lipogenesis, and glucose uptake (Achari & Jain, 2017). For AdipoR2, APPL1/2 is responsible for the activation of a pathway that leads to the enhancement of β‐oxidation through Acyl‐CoA oxidase (ACO), a mechanism different from that recruited by AdipoR1. ADPN preferential binding to AdipoR2 is also able to activate p38 MAPK and the PPARα pathway, which are involved in the regulation of lipid metabolism and energy homeostasis (Ajuwon & Spurlock, 2005a; Yamauchi et al., 2014). Experiments conducted on the murine myoblast cell line C2C12 showed that APPL‐1 mediates ADPN stimulatory effect on p38 MAPK (Xin et al., 2011), and, in pig adipocytes, ADPN can be a local regulator of adipose tissue inflammation, through its regulation of the nuclear factor kappa‐light‐chain‐enhancer of activated B cells (NFKβ) and PPARγ2 (Ajuwon & Spurlock, 2005a). Interestingly, ADPN was previously shown to induce the expression of PPARγ2 also in murine adipose tissue (Ziemke & Mantzoros, 2010).

**FIGURE 1:**
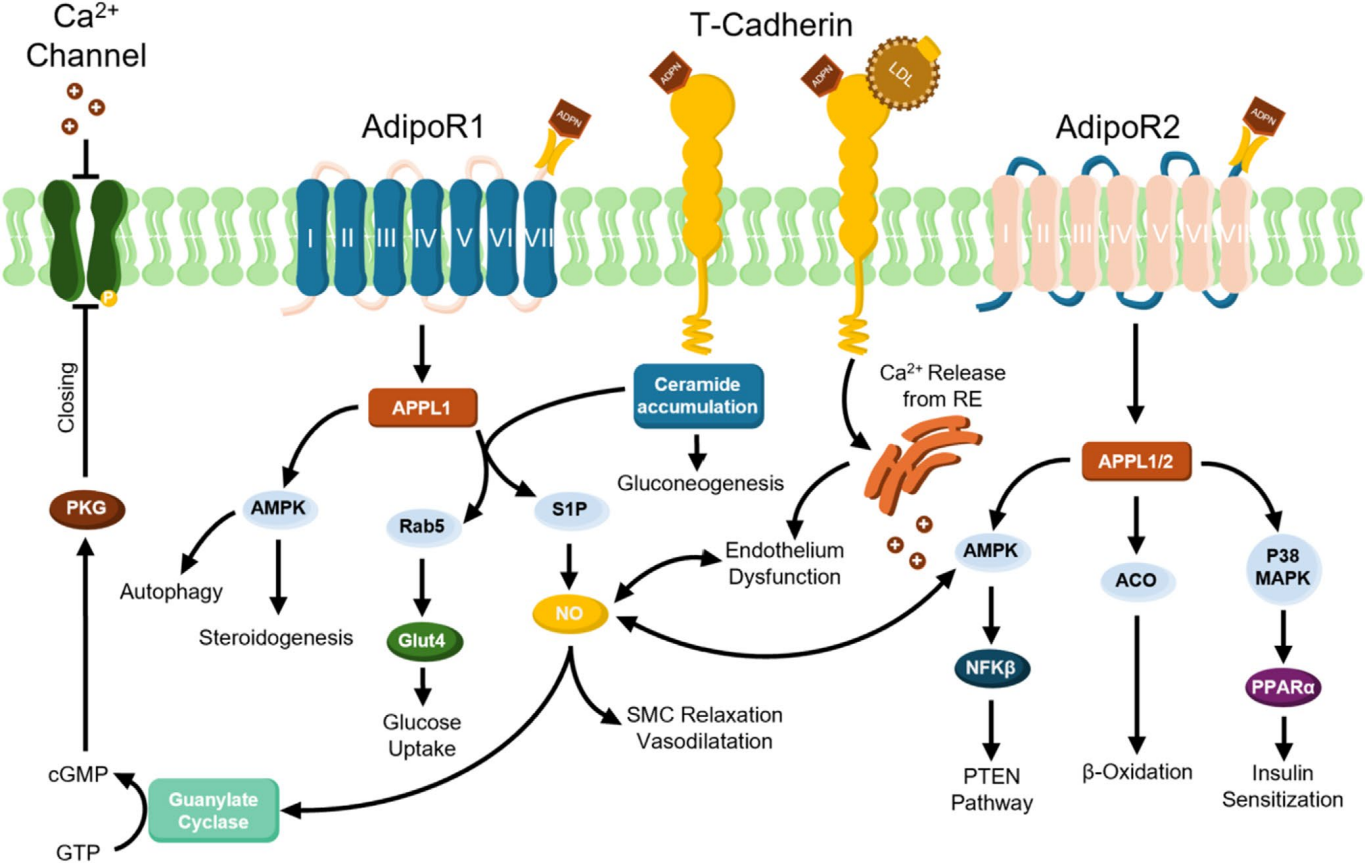
Adiponectin receptors and signaling pathways engaged after the binding with the hormone. APPL1 from AdipoR1 shows a crosstalk with the analogue pathway from the insulin receptor, leading to several effects on metabolism such as an increase in protein and glycogen synthesis, gluconeogenesis, lipogenesis, and glucose uptake (Achari & Jain, 2017). For AdipoR2, APPL1/2 activates a pathway that leads to the enhancement of β‐oxidation through Acyl‐CoA oxidase (ACO), a mechanism different from that recruited by AdipoR1. ADPN preferential binding to AdipoR2 is also able to activate p38 MAPK and the PPARα pathway, involved in the regulation of lipid metabolism and energy homeostasis (Ajuwon et al., 2005b; Yamauchi et al., 2014). AMPK activation, following the binding of ADPN with AdipoR2, besides modulating NO synthesis, also triggers nuclear factor kappa‐light‐chain‐enhancer of activated B cells (NFKβ) and Phosphatase and tensin homolog (PTEN) pathway: PTEN acts as a tumor suppressor gene thanks to the action of its phosphatase protein product, which is involved in the regulation of the cell cycle and is a target of several anticancer drugs (Chu & Tarnawski, 2024). ACO, acyl‐CoA oxidase; AdipoR, adiponectin receptor; ADPN, adiponectin; AMPK, 5' AMP‐activated protein kinase; APPL, adaptor protein, phosphotyrosine interacting with PH domain and leucine zipper; cGMP, cyclic guanosine monophosphate; Glut4, glucose transporter type 4; GTP, guanosine‐5′‐triphosphate; LDL, low‐density lipoprotein; NFKβ, nuclear factor kappa‐light‐chain‐enhancer of activated B cells; P38 MAPK, p38 mitogen‐activated protein kinases; PKG, protein kinase G; PPARα, peroxisome proliferator‐activated receptor alpha; PTEN, phosphatase and tensin homolog; Rab5, Ras‐related protein Rab‐5; RE, endoplasmic reticulum; S1P, sphingosine‐1‐phosphate; SMC, smooth muscle cell.

Finally, AMPK activation, following the binding of ADPN with AdipoR2, besides modulating NO synthesis, also triggers NFKβ and Phosphatase and tensin homolog (PTEN) pathway. PTEN acts as a tumor suppressor gene thanks to the action of its phosphatase protein product, which is involved in the regulation of the cell cycle and is a target of several anticancer drugs (Chu & Tarnawski, 2024). A scheme presenting ADPN receptors and the main signaling pathways activated is depicted in Figure 1.

### 3.1 ADPN and feeding behaviors

**FIGURE 2:**
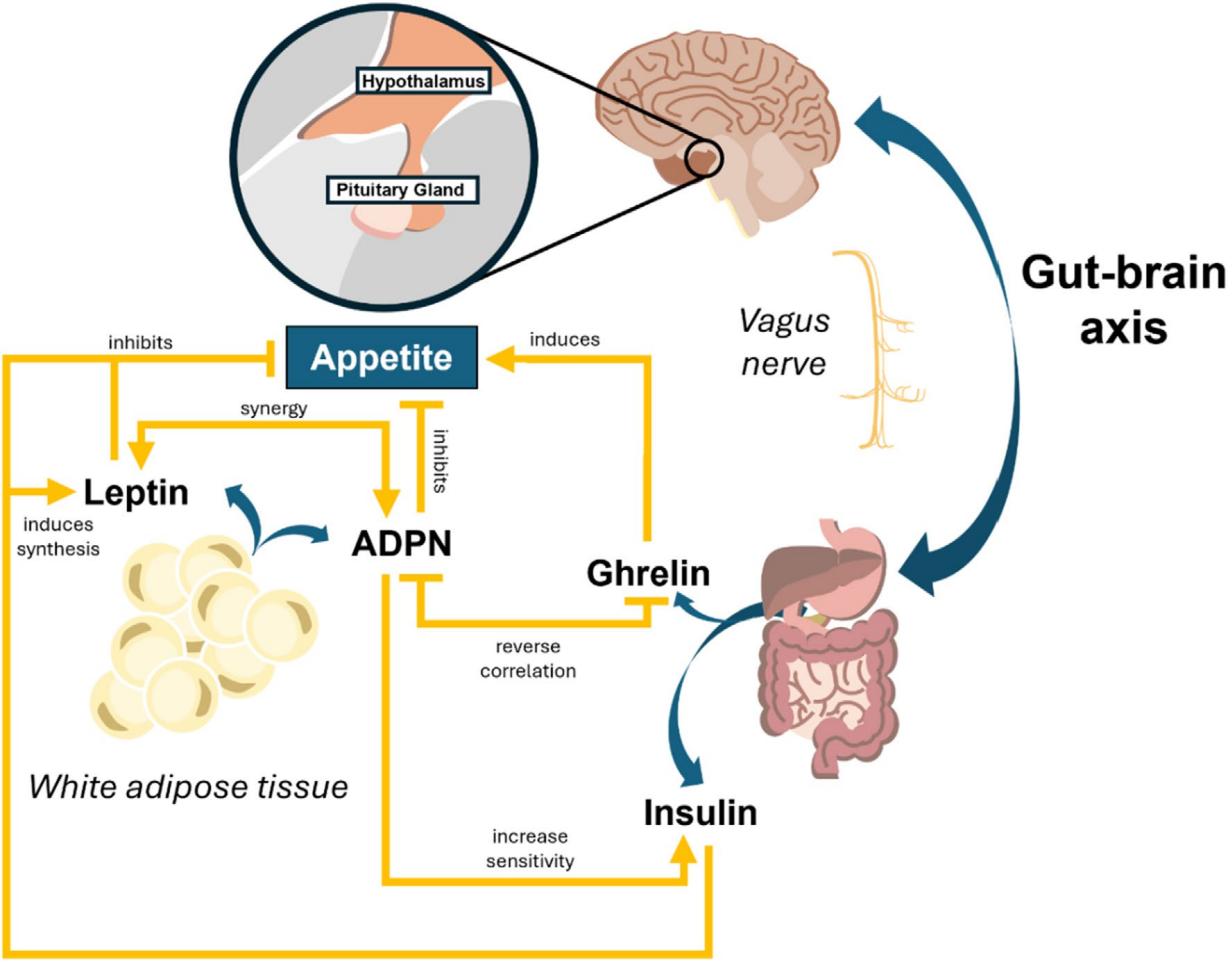
Control of feeding behaviors. Scheme illustrating the neural and hormonal interplay among the gastrointestinal tract, the brain and the white adipose tissue in the control of appetite. The vagus nerve is the main conduit for bidirectional communication between the brain and gastrointestinal tract functions. The efferent fibers running in the vagus nerve affect smooth muscle cell motility and cell secretion. The afferent vagal fibers respond to gastric and gut mechanoreceptors, gut peptides and neurotransmitters. At the hypothalamus level (inset) the key neurons controlling the homeostatic cycle of hunger and satiety are located. Adipokines released by the white adipose tissue (leptin and adiponectin‐ADPN) cooperate in synergy with insulin to inhibit appetite. In contrast, ghrelin produced by the stomach stimulates food intake.

The control of feeding behaviors involves a complex interplay between peripheral tissues and different brain regions (Cifuentes & Acosta, 2022). Among these, the hypothalamus plays a key role, but the brainstem, limbic system, and prefrontal cortex also take part in the cooperation by integrating neural, hormonal, and environmental signals to regulate hunger, satiety, and food intake (Morton et al., 2006). In particular, neurons in the arcuate nucleus (ARC) of the hypothalamus produce neuropeptides that either stimulate (e.g., neuropeptide Y [NPY] and agouti‐related peptide [AgRP]) or suppress appetite (e.g., pro‐opiomelanocortin [POMC], cocaine‐ and amphetamine‐regulated transcript [CART]). Moreover, the mesolimbic system and prefrontal cortex (reward system) are involved in the hedonic aspects of eating, influencing food preferences and motivation to eat (Liu & Kanoski, 2018). A number of nervous, hormonal, and metabolic peripheral mechanisms take part in this interplay (summarized in Figure 2).

For instance, gastric distention caused by meal ingestion activates vagal afferents, which send satiety signals from the stomach to the brain. The hormonal regulation involves a composite and still not completely understood system of feedback between the brain and peripheral organs, including white adipose tissue and the gastrointestinal tract, to ensure that energy intake aligns with the body's needs, maintaining overall energy balance. Peptides like leptin, produced by white adipose tissue, and insulin, produced by pancreatic beta‐cells, circulate in the bloodstream and act on the hypothalamus to convey information about energy stores, thereby modulating appetite (inducing satiation) and energy expenditure (Valassi et al., 2008). The stomach releases ghrelin, which stimulates appetite, whereas gut hormones such as peptide YY (PYY) and cholecystokinin (CCK) promote satiety. These hormones communicate with the brain via the vagus nerve and bloodstream to regulate meal size and frequency (York, 1999) and, potentially, food preference as well (Disse et al., 2010).

Although not a primary appetite‐regulating hormone, ADPN may influence appetite as well. In a recent study on a goldfish model, ADPN has been recognized as a novel satiety factor where feeding can trigger ADPN central and peripheral signals (Zheng et al., 2023). Indeed, ADPN may affect appetite in various ways: it may either exert a central action, although interacting with other hormones, or act on peripheral organs, modulating energy balance. First, ADPN may exert a central action since it can cross the blood–brain barrier (BBB) and can directly modulate hypothalamic pathways (Hirschberg, 1998; Shklyaev et al., 2021; Stanley et al., 2005). In particular, it interacts with specific nuclei in the central nervous system through its receptors: AdipoR1 and AdipoR2 are, in fact, expressed in the hypothalamus and the nucleus of the solitary tract (NTS). In the ARC nucleus, ADPN activates AMPK and promotes food intake as evaluated in refed rodents (Kubota et al., 2007). In the NTS, ADPN interacts with AdipoR1 and AdipoR2 to regulate autonomic functions, such as blood pressure control (Hoyda et al., 2009). ADPN has also been shown to modulate dopamine‐related reward mechanisms (Klockars et al., 2021), suggesting its involvement also in non‐homeostatic feeding behaviors.

Nonetheless, ADPN can also influence the central nervous system indirectly. ADPN can be found in cerebrospinal fluid, and its concentration increases following intravenous administration. This suggests a crosstalk between central and peripheral tissues, promoting the hypothesis that ADPN contributes to body–brain interaction, in particular for appetite regulation and energy homeostasis. Again, one way ADPN may achieve body–brain interaction is by acting on peripheral nerves, such as the vagus (Ahima & Antwi, 2008).

Several studies evaluated the endocrinological aspects of appetite regulation, highlighting ADPN, alongside leptin and ghrelin, as a key hormonal player in this process (Funcke & Scherer, 2019; Meier & Gressner, 2004). In this regard, the interaction of ADPN with leptin and ghrelin has been previously extensively studied (Yildiz et al., 2004): with evidence showing that while leptin suppresses hunger and ghrelin promotes hunger, ADPN appears to enhance insulin sensitivity (Figure 2), modulating energy balance rather than directly controlling hunger. By increasing insulin sensitivity and fatty acid oxidation (Achari & Jain, 2017), ADPN plays a role in glucose homeostasis, indirectly affecting feeding behaviors.

Moreover, ADPN can exert several important peripheral effects that influence various physiological processes, particularly in tissues involved in metabolism and energy balance, such as skeletal muscle (Liu & Sweeney, 2014), liver (Combs & Marliss, 2014), cardiac (Ding et al., 2012), or adipose tissues (Moschen et al., 2012). This concerted action is crucial for maintaining energy balance, insulin sensitivity, reducing inflammation, and promoting fatty acid oxidation. ADPN may also influence gut microbiota composition (Polito, Di Meo, et al., 2020; Polito, Monda, et al., 2020), once more indirectly affecting satiety signals.

Notably, the growing interest in the appetite regulation function of ADPN is also supported by the observations that disruptions in ADPN levels contribute to metabolic imbalances and altered feeding patterns. Although a growing amount of evidence has been provided, the role of ADPN in the gut–brain axis and satiety remains controversial due to conflicting results (see Tang et al., 2021 for a review on this topic). A number of studies (Zheng et al., 2023) suggest that ADPN has an anorexigenic action, activating AMPK in the hypothalamus, leading to increased energy expenditure. Moreover, ADPN increases glucagon‐like peptide‐1 (GLP‐1), PYY, and CCK secretion, all of which reduce hunger. Indeed, central administration of ADPN decreases food intake in rodents (Suyama et al., 2017).

However, other mechanisms of action suggest an orexigenic effect for ADPN, especially with regard to its interaction with ghrelin, where conflicting findings have been reported (some studies suggest inhibition, others suggest stimulation) (van Egmond et al., 2023; Yildiz et al., 2004). ADPN levels are inversely correlated with leptin, suggesting a potential compensatory mechanism in energy balance that could increase feeding in certain conditions. Possible explanations for the contradiction may be related to feeding status, the content of glucose in the cerebrospinal fluid, and the degree of fatness (Tang et al., 2021) as well as the specific ADPN isoforms tested since total ADPN, HMW, and LMW might have different metabolic effects (Lara‐Castro et al., 2006). In addition, different concentrations may induce different feeding responses, and finally, species‐specific differences may occur.

## 4 EFFECTS ON GASTRIC SMOOTH MUSCLE

The stomach is a key organ of the digestive system, working as a storage chamber for ingested food and allowing its progressive breakdown and passage into the duodenum. In particular, the proximal stomach smooth muscle tone is essential for maintaining the suitable organ's shape to control adaptive relaxation (Mayr et al., 2024), intragastric pressure and, as stated above, sending peripheral satiety signals to the hypothalamus. In addition, it contributes to proper gastric motility to drive peristaltic movements. These functions lead the stomach to be an interesting candidate as a potential target organ of ADPN, for all physio‐pathological conditions where this hormone is involved. Understanding the regulatory mechanisms of gastric excitability, tone, and mechanical response is essential for diagnosing and treating various gastrointestinal disorders associated with altered gastric motility.

Even if the ADPN mechanism of action on the stomach is not yet fully understood, some preclinical investigations and studies on animal models offer attractive insights that can be interesting and promising for further analyses. Recent scientific research (Chang et al., 2020) highlights a widespread role of ADPN in gastrointestinal health, where it has been shown to protect against chronic inflammation‐induced colon cancer (Wei et al., 2005) and to influence immune homeostasis in the context of intestinal inflammation (Weidinger et al., 2018) Specifically, ADPN causes numerous effects on the stomach, influencing several physio‐pathological functions, such as gastric motility, appetite regulation, and inflammation (Choi et al., 2020), supporting a potential role in regulating food intake (Idrizaj, Garella, Nistri, et al., 2020; Idrizaj, Garella, Squecco, & Baccari, 2020), and underscoring the multifaceted contributions of this hormone to gastric physiology and pathology.

### 4.1 Functional modifications induced by ADPN

ADPN, through the activation of AdipoR1 and AdipoR2, can modulate murine gastric muscle excitability, tone, and motility, which are all essential for effective adaptive relaxation, organ emptying, and proper digestion (Garella, Bernacchioni, et al., 2023). In particular, our research group showed that the addition of ADPN to murine gastric fundus muscle strips determines a progressive decay of the basal tension and enhances the size of the neurally‐induced relaxant responses, demonstrating the hormone's ability to exert a pro‐relaxing effect as mediated by the NO pathway (Idrizaj et al., 2018). Following studies showed that ADPN strongly influences the bioelectrical properties of smooth muscle cells, inducing several plasma membrane changes that hinder cellular excitability. These results, once verified in humans, pose a striking question from a possible translational point of view, since eventual alterations in smooth muscle cell excitability, consequently accompanied by altered muscle tone, can lead to significant gastric dysfunctions of its mechanical activity.

More specifically, our research group recently described that—among other modifications in smooth muscle cells—ADPN induces an increase in cell capacitance (Garella, Cassioli, et al., 2023). This is the electrophysiological parameter related to cell surface extension suggestive of a possible change of shape (i.e., elongation) of smooth muscle cells, a change which may be secondary to altered arrangements of the contractile machinery. In addition, we also observed that ADPN systematically causes membrane potential hyperpolarization (Idrizaj et al., 2019), and an increase in the amplitude of K^+^ currents (Garella, Cassioli, et al., 2023; Idrizaj, Garella, Nistri, et al., 2020; Idrizaj, Garella, Squecco, & Baccari, 2020), suggesting impaired ion channels' function. Such effects exhibit an interplay with each other, shifting the membrane potential downwards from the threshold of action potential onset. This can hinder electromechanical coupling, counteracting contractile activation, favoring a more relaxed condition of the cells, leading to a decreased gastric tone, delayed gastric emptying, uncoordinated or ineffective peristaltic contractions and, eventually, gastroparesis.

Furthermore, our research group also reported that ADPN causes a reduction of calcium current (I_Ca_) amplitude (Idrizaj, Garella, Nistri, et al., 2020; Idrizaj, Garella, Squecco, & Baccari, 2020). Voltage‐gated Ca^2+^ channels are sensitive to changes in membrane potential and, once activated, these channels allow a calcium influx that increases internal Ca^2+^ concentration. Calcium influx is in turn useful to promote contractile apparatus activation, as depolarization further proceeds. The membrane hyperpolarization induced by ADPN basically hinders this event. After ADPN addition, a functional reduction of I_Ca_ was observed in the ex vivo murine gastric fundus. This suggests a clear inhibitory effect on Ca^2+^ influx through voltage‐gated Ca^2+^ channels and further supports the influence of this adipokine in hindering the electromechanical coupling of smooth muscle cells (Idrizaj, Garella, Nistri, et al., 2020; Idrizaj, Garella, Squecco, & Baccari, 2020).

Additionally, gastric unitary smooth muscle functionality is also strictly related to inter‐cell electrical coupling. This coupling allows for the electrical signals to run on the membrane of a smooth muscle cell and to be rapidly transmitted to adjacent cells by means of the gap junctions, thus leading to the excitation of the whole muscle layer (Cousins et al., 2003; Klemm et al., 2020). In this regard, ADPN was also shown to hinder the electrical coupling mediated by the Connexin 43 protein (Garella, Cassioli, et al., 2023). These findings once again support the modulatory role of ADPN in lessening the contractile response of gastric fundus.

In brief, this line of research supports the hypothesis that ion channels and membrane proteins represent molecular targets of ADPN, leading to gastric smooth muscle hyperpolarization and reduced muscle tone. Confirmation of these data in human samples (i.e., from gastric surgical specimens) could be promising for future translational purposes.

### 4.2 Morphological modifications induced by ADPN

Despite the fact that several studies have demonstrated an important functional role for ADPN at the gastric level, highlighting its pro‐relaxant effect, with potential implications for the control of appetite and hunger‐satiety, only a few researchers investigated the related morphological alterations underlying these processes. This field of research, however, can be especially of interest in order to substantiate the effect induced by this hormone and to contribute to identifying the cellular and molecular mechanisms underpinning its action. Our previous basic research conducted on murine tissue showed that ADPN alters the structural organization of smooth muscle contractile filaments, promoting a more relaxed gastric state (Garella, Bernacchioni, et al., 2023; Garella, Cassioli, et al., 2023). Confocal immunofluorescence analysis revealed that ADPN induces the expression of α‐smooth muscle actin (sma), the most abundant actin isoform composing thin myofilaments.

Ultrastructural analysis also showed that ADPN induces a diverging aggregation of thin actin filaments. Indeed, transmission electron microscopy showed that ADPN‐treated cells exhibited a slightly looser network of contractile filaments, suggestive of a more relaxed state. In parallel, ADPN‐treated muscular samples exhibit a lower expression of the phosphorylated form of myosin light chain 2 (p‐MLC2). Since p‐MLC2 is required for smooth muscle cell contraction, this observation corroborates the capability of ADPN to favor smooth muscle cell relaxation. Notably, the effect of ADPN on the morphological features of the smooth muscle cells and on their biophysical membrane properties was mediated by SK2‐triggered signaling path (Garella, Bernacchioni, et al., 2023). These findings, especially if verified in human samples, could have important implications for conditions affecting gastric motility and tone.

To move towards insight over non‐physiological conditions, it has been suggested that ADPN has a role in gastric tumorigenic transformation. Although no specific studies focused on morphological alterations of the contractile apparatus as induced by ADPN, evidence has been reported on specific structural alterations as related to AdipoR1/R2. A recent clinical study analyzed ADPN's implication in gastric cancer progression evaluating the relation between AdipoR1/R2 expression and gastric intestinal metaplasia (Ayyildiz et al., 2021). By an immunohistochemical evaluation, a significantly lower AdipoR1/R2 expression in patients with gastric cancers was observed, as compared to patients with gastric intestinal metaplasia, suggesting that ADPN may have a role in gastric cancer progression as mediated by AdipoR1/R2 receptors. As further confirmation of the protective role of ADPN in cancer, Larsson and colleagues recently proposed that ADPN plays an important role in mediating the link between overall fat deposits and cancer risk (Larsson et al., 2022). In particular, ADPN binding to the specific receptors AdipoR1/R2 induces AMPK activation, which is involved in several signaling pathways correlated with tumor growth inhibition.

In summary, ADPN is a hormone involved in both physiological and nonphysiological processes, and its activation of peculiar signaling pathways could be correlated with the onset of morphological modifications. Therefore, investigating the possible effects of ADPN on the stomach, from a functional but also a morphological point of view, could be of considerable importance to better understand its role in maintaining the homeostasis of the hunger‐satiety cycle, as well as in preventing metaplasia or neoplasia.

However, one key aspect of the translation of the above proposed mechanisms relies, first of all, on the confirmation of the presence of ADPN receptors in human samples. In this regard, evidence of AdipoR1 and AdipoR2 expression has been provided at the RNA level in different gastric regions, including the gastric fundus (GTEx Consortium, 2020). Moreover, real‐time PCR, Western blotting, and immunohistochemical staining revealed the presence of the receptors in some districts of the human gastrointestinal tract, such as normal colon epithelial and colon cancer cells (Yoneda et al., 2008), or in cells of human gastric tissue (both in physiological conditions, and as expressed in metaplastic tissue) (Lin et al., 2021).

## 5 POTENTIAL TRANSLATIONAL PERSPECTIVES

To properly bridge the bulk of findings from preclinical studies on rodent models to human clinical contexts, it is mandatory to compare in advance ADPN's functional roles, circulating concentrations, and receptor expression patterns between these species. As stated above (Section 2. Structure and functions of ADPN), while some differences have been shown in circulating concentrations of ADPN between the two species, ADPN seems to share the same receptors, which are similarly distributed across tissues, and seem to activate the same signaling pathways and serve the similar fundamental roles in glucose and lipid metabolism, insulin sensitivity, or anti‐inflammatory action, in both mice and humans (Tilg et al., 2024). In this light, we here discuss some widespread clinical conditions where the use of ADPN could be potentially helpful.

### 5.1 Eating disorders

ADPN has recently gained attention for its potential role in eating disorders due to its influence on metabolism, energy regulation, and inflammation, all of which are crucial in conditions such as AN, BN, and BED (Dani et al., 2024). Given the significant overlap between metabolic comorbidities, or even complications, in patients with eating disorders, ADPN may not be fully employed as a diagnostic biomarker capable of discriminating against medical conditions, thus reducing the potential to discriminate between full‐blown psychiatric conditions and medical conditions mimicking symptoms of eating disorders. In fact, as previously mentioned, ADPN has been observed as decreased among different conditions, such as BN, BED, and obesity. However, ADPN may aid in distinguishing between AN and BN (being increased in AN, and decreased in BN) or BN and BED (being more strongly reduced in BED), addressing the ongoing debate regarding the limitations of diagnostic differentials imposed by weight thresholds (see Table 1).

**TABLE 1:** Role of adiponectin (ADPN) in eating disorders.

Eating disorder	ADPN levels	Key mechanisms	Potential therapeutic implications
AN	Elevated	Adaptive response to caloric restriction. Enhances fatty acid oxidation and insulin sensitivity. Potential role in appetite regulation (interacts with leptin and ghrelin). Anti‐inflammatory effects (Robinson et al., 2011). Possible role in stress response (Fantuzzi, 2014). High ADPN could blunt hunger signals despite low leptin levels, promoting food avoidance. (Modan‐Moses et al., 2007). ADPN interacts with ghrelin to modulate appetite suppression, possibly by disrupting gut–brain axis regulation.	ADPN as a staging biomarker or predictor of treatment response. Potential therapeutic use of ADPN analogues for targeting metabolic and psychological symptoms.
BN	Moderately Reduced	Associated with increased BMI and metabolic syndrome. Linked to insulin resistance and poor glucose metabolism. Involved in inflammation and hunger/satiety dysregulation. May contribute to loss of control over food intake and binge eating episodes (interaction with leptin and ghrelin).	ADPN analogues to improve metabolic health and reduce inflammation. Potential role in restoring hunger and satiety signals.
BED	Strongly Reduced	Associated with obesity, metabolic syndrome, and insulin resistance, hyperglycemia. Impaired fatty acid oxidation and glucose metabolism. Increased pro‐inflammatory status (Choi et al., 2020). Increased hunger, loss of control over food intake, disruption of satiety signals (leptin and ghrelin). Low ADPN may be linked to leptin resistance (Tural et al., 2023), disrupting satiety regulation. Low ADPN disrupts ghrelin signaling, leading to increased hunger and binge eating episodes. (Tagami et al., 2004).	ADPN analogues as a treatment for metabolic dysfunction. Potential role as a biomarker for disease severity and treatment response.

Abbreviations: AN, anorexia nervosa; ADPN, adiponectin; BED, binge eating disorder; BMI, body mass index; BN, bulimia nervosa.

Although ADPN has not yet shown significant potential as a diagnostic biomarker for eating disorders (i.e., not stratifying patients according to diagnostic severity), ADPN may serve as a prognostic biomarker, providing clinical guidance for these patients, potentially informing on the metabolic risk of single patients. This potential is urgently needed, considering the significant neglected needs in this clinical population with adverse cardiovascular events being main contributors to both morbidity and mortality (Sachs et al., 2016).

The lack of potential to stratify patients according to diagnostic severity, however, needs to be properly conceptualized. In fact, unfortunately, although current diagnostic criteria posit BMI as the indicator of severity (e.g., for AN), empirical results have found BMI not adequately capturing the complexity of eating disorders (see for example, Dang et al., 2023). Recent studies have highlighted that body composition (lean vs. fat mass) may better be associated with cardiac functioning, and thus treatment response, that is, weight restoration in AN (Tarchi, Garella, et al., 2024; Tarchi et al., 2025). However, body composition analysis requires specific training and equipment. A biomarker linked to both fat mass loss and cardiovascular health has the potential to significantly improve clinical outcomes in eating disorders, providing clinical guidance as a potential prognostic biomarker. Moreover, patients frequently transition between different diagnoses (e.g., AN and BN, or even BED) (Castellini et al., 2011). ADPN may then serve as an individual‐level biomarker, monitoring diagnostic cross‐over.

Although ADPN may not reach a stringent demarcation between clinical and nonclinical groups, it may capture the transition between pre‐clinical and clinical conditions, a crucial need in eating disorders. In fact, a wide range of individuals may report over‐eating and attempt to restrict eating behaviors. For instance, 56% of young women and up to 31% of young men between the ages of 14 and 18 may engage in restrictive eating behaviors (Croll et al., 2002). In parallel, sub‐threshold or prodromal symptoms are increasingly recognized as clinically relevant (Le Grange & Loeb, 2007) and may represent early manifestations of disorders at a higher risk of chronicity and relapse, at least indirectly, as contributors to increased untreated and increased undiagnosed time (Le Grange & Loeb, 2007). ADPN may then be investigated in relation to the early detection of dysfunctional eating behaviors, or as a predictor of diagnostic progression, improving early detection and ultimately addressing significant neglected needs in this clinical population (Bulik, 2021).

While the exact mechanisms are still under study, research thus suggests that ADPN might play a multifaceted role in eating e disorders and may offer insights over a variety of clinical uses (Table 1).

#### 5.1.1 ADPN in AN

ADPN plays a complex role in AN, where it is involved in the metabolic and physiological adaptations to the extreme calorie restriction typical of this eating disorder. The hormone levels are often paradoxically elevated in individuals with AN, despite the severe weight loss and low body fat percentage (Arita et al., 1999; Iwahashi et al., 2003; Tural et al., 2023). This is unusual because adipokines are typically produced by adipose tissue, and their levels normally directly correlate with fat mass. The high levels of ADPN in AN could serve as a compensatory mechanism aimed to maximize energy efficiency, promoting fatty acid oxidation and improving insulin sensitivity. In fact, a shift towards fatty acid oxidation is commonly observed in patients with AN (Yamauchi et al., 2001, 2002): the body attempts to preserve glucose for essential functions, such as brain activity, while relying on fatty acids for energy in peripheral tissues. In a context in which extreme caloric deficit is crucial, ADPN thus helps the body cope with prolonged periods of low caloric intake by shifting energy use towards stored fats. Hyper‐adiponectinemia might then buffer the energy deficit, but at the same time, may also make recovery more difficult by promoting blunted energy expenditure even in a state of malnutrition, inhibiting anabolism. In summary, ADPN in AN may first exert an adaptive function, but later risks favoring maladaptive mechanisms of maintenance. This effect of ADPN, first adaptive, then maladaptive, may be postulated considering that a similar mechanism of action has been described for other metabolically relevant factors in AN (Tarchi, Merola, et al., 2024).

However, a recent cross‐sectional clinical study from our group revealed a lack of clear correlation between ADPN plasma levels and psychopathological severity in AN, thus discouraging the potential use of ADPN as a valid diagnostic biomarker in AN (Garella, Cassioli, et al., 2023). Nonetheless, considering how ADPN may exert adaptive or maladaptive functions in light of serum concentrations, duration of illness, and thus disease course, ADPN could better serve as a staging biomarker or as a predictor of treatment response, indicating which patients are at the highest risk of no treatment response, relapse, or unsatisfactory recovery (Table 2).

**TABLE 2:** ADPN as a biomarker in eating disorders.

Application	Potential use
Diagnostic biomarker	Measuring serum levels of ADPN may help in the diagnostic differential between AN and BN. This potential derives from the possibility of inferring fat mass loss over a certain threshold, as associated with ADPN serum levels. In fact, the key diagnostic differential between AN and BN is given by the Criterion A of the DSM‐5/DSM‐5‐TR (significant low body weight). However, which specific threshold of weight may differentiate AN and BN is a point of debate, also considering potential ethnic confounders in BMI thresholds (Deurenberg et al., 2002).
Staging biomarker	Monitoring ADPN levels may help assess disease stage and progression in AN, reaching a better marker of severity than BMI alone (Dang et al., 2023). Moreover, ADPN may screen for the risk of cardiovascular complications/comorbidities, a significant contribution to increased morbidity and mortality in eating disorders (Sachs et al., 2016).
Predictor of treatment response	ADPN levels could indicate which patients are at high risk for relapse or poor recovery, in AN, BN, or BED. For instance, ADPN may exhibit this potential as it helps screening for severe fat loss or muscle cachexia. Detecting muscle cachexia may be crucial, as it is also associated with reduced cardiac functioning, a risk factor for lower weight restoration in AN (Tarchi, Garella, et al., 2024; Tarchi, Merola, et al., 2024). Moreover, ADPN regulates ceramide metabolism, which have been implicated in depressive disorders (Bernal‐Vega et al., 2023; Tomasik et al., 2024). Depressive disorders represent key comorbidities increasing the risk of lower treatment response in eating disorders (Cassioli et al., 2023). ADPN may thus detect patients at high‐risk of low treatment response or relapse.
Metabolic health indicator	ADPN, a key regulator of food intake and metabolic pathways, could help assess metabolic dysfunction in BN and BED, guiding treatment and helping managing medical comorbidities (Chaurasia & Summers, 2015).

Abbreviations: ADPN, adiponectin; AN, anorexia nervosa; BED, binge eating disorder; BMI, body mass index; BN, bulimia nervosa; DSM, diagnostic and statistical manual of mental disorders.

In fact, elevated levels of ADPN commonly observed in AN could interact with decreased leptin or increased ghrelin in a complex interaction which future studies may aim to further investigate.

In regards to its anti‐inflammatory properties, ADPN could play a role in mitigating the systemic inflammation commonly associated with severe malnutrition in AN (Robinson et al., 2011). Additionally, ADPN could regulate the body's response to stress in AN, as some authors suggested that elevated levels of ADPN could help reduce stress‐induced inflammation (Fantuzzi, 2014), although the exact mechanism remains unclear. Moreover, a current meta‐analysis indicated that glucose metabolism and inflammatory molecules may explain the relationship between AN and ADPN serum levels (Tural & Iosifescu, 2022).

Peptide analogues have begun to accrue significant interest in pharmacological research (Di Santo et al., 2025). However, research in psychiatry in general and eating disorders more specifically is arguably lagging behind other fields of research. In fact, while leptin analogues have since been investigated in AN (Milos et al., 2020), the potential for other analogues remains unexplored, including ADPN. However, ADPN analogues or receptor antagonists could find a potential use in the treatment of eating disorders, as schematized in Table 3. ADPN analogues could positively modulate energy metabolism and appetite in AN, while also reducing the risk of atherosclerosis and adverse cardiac events. Additionally, ADPN analogues may modulate ceramide synthesis and thus impact mood regulation (Dinoff et al., 2017). Although to our knowledge there are currently no studies regarding this use in AN, research is active in the field, with specific molecules being developed (e.g., AdipoRon—a synthetic small‐molecule agonist of the AdipoR1). These developed molecules act on related metabolic pathways while also modulating appetite and energy metabolism. Moreover, considering the high burden of depressive symptoms in AN, a possible use of ADPN analogues as antidepressant molecules could also be taken into account (Bhat et al., 2020), in light of ADPN's anti‐inflammatory effects and in light of ADPN's improvement of neurogenesis (You et al., 2021).

**TABLE 3:** Potential therapeutic strategies targeting ADPN in eating disorders.

Therapeutic strategy	Mechanism	Potential benefit
ADPN Analogues (e.g., AdipoRon)	Selective agonism or antagonism of AdipoR1, AdipoR2 (AMPK and PPARα pathways; Okada‐Iwabu et al., 2013)	Agonism: improves insulin resistance and glucose metabolism; potential appetite modulation in AN, BN, and BED, promotion of neurogenesis, antidepressive effects Antagonism: Counteract hyper‐adiponectinemia in AN.
Anti‐inflammatory Approaches	Targeting peripheral ADPN receptors to inhibit inflammation pathways.	Agonism: May reduce systemic inflammation associated with AN, BN, and BED.
Exercise and Lifestyle Interventions	Modulatory (pro‐homeostatic) effect on ADPN levels.	Can improve ADPN in hypo‐adiponectimia, promote a return to physiological levels in hyper‐adiponectinemia. Can improve metabolic health and psychological well‐being in BED, and in selected patients with BN or AN.
Pharmacological Modulation of ADPN Pathways	Enhancing ADPN secretion or function	Agonism: potential role in normalizing appetite regulation and metabolic balance.

Abbreviations: ADPN, adiponectin; AdipoR1, adiponectin receptor 1; AdipoR2, adiponectin receptor 2; AN, anorexia nervosa; BN, bulimia nervosa; BED, binge eating disorder; AMPK, 5'‐AMP‐activated protein kinase; PPARα, peroxisome proliferator‐activated receptor alpha.

In summary, normalizing ADPN levels during recovery from AN could be beneficial in restoring metabolic balance and possibly reducing the risk of relapse (Haines, 2023).

#### 5.1.2 ADPN in BN

ADPN plays a notable but less well‐understood role in BN compared to that in AN. While BN has been described as further characterized by a higher impulse dyscontrol and a higher reliance on compensatory behaviors, these differences now seem to play a minor role in comparison to the diagnostic differential given by body mass index (BMI) and thus metabolic factors (Hübel et al., 2021; Puttevils et al., 2021; Watson et al., 2013). Unlike AN, individuals with BN often exhibit lower levels of ADPN (Table 1), especially in cases where obesity or metabolic syndrome is also present (Tural et al., 2023).

Previous studies show that patients with BN, especially those with a higher BMI, tend to have reduced circulating ADPN levels, generally associated with insulin resistance and poor glucose metabolism (Caselli, 2014; Yadav et al., 2013). In addition, patients with BN show increased inflammation (Dani et al., 2024), which is linked to both metabolic disorders and psychological stress (Piao et al., 2017) and dysregulation in hunger and satiety signals (Tang et al., 2021).

As mentioned above, even if ADPN may not be considered a direct appetite‐regulating peptide, its involvement in feeding behavior is strictly related to its interaction with other hormones, such as leptin and ghrelin (Figure 2).

Restoring or increasing ADPN levels in individuals with BN through ADPN analogues could offer potential therapeutic benefits: by increasing the hormone levels, it might be possible to reduce the chronic inflammation observed in bulimic patients, which could improve both metabolic and psychological outcomes. Moreover, elevating ADPN analogues could improve insulin sensitivity, potentially helping to restore normal glucose metabolism and reducing cravings for high‐sugar foods that often drive binge episodes. Enhancing ADPN levels could also improve the interaction with leptin and ghrelin, re‐establishing physiological hunger and satiety signals and thus potentially reducing the burden of binge eating episodes. Targeting ADPN could thus be a valuable strategy for addressing neglected needs in both physical and psychological domains for BN (Feng et al., 2023).

#### 5.1.3 ADPN in BED

ADPN plays a complex role also in BED, a condition characterized by recurrent episodes of binge eating without compensatory behaviors as seen in AN or BN (Tural et al., 2023). Individuals with BED commonly suffer from metabolic disturbances, pro‐inflammatory status, obesity, and insulin resistance, conditions in which ADPN is heavily involved (Dani et al., 2024).

Research consistently shows that individuals with BED, especially those who are overweight or obese, tend to have low circulating levels of ADPN (Khalil & El Hachem, 2014; Tural et al., 2023), which may be associated with a range of negative outcomes that contribute to binge eating behaviors and metabolic dysregulation (Table 1). Lower levels of the hormone can lead to hyperglycemia and insulin resistance, which can drive hunger and loss of control over food intake. Low ADPN is also linked to increased levels of pro‐inflammatory cytokines. This chronic inflammation can affect not only physical health but also mental health, exacerbating stress and emotional triggers for binge eating (Razzoli et al., 2017).

In BED, leptin resistance often occurs (Liu et al., 2018; Obradovic et al., 2021), particularly in individuals with obesity. ADPN can modulate leptin sensitivity, and low levels of the hormone may impair the normal leptin‐mediated satiety signals. As a result, individuals may not experience physiological satiety signals, leading to prandial overconsumption, as well as nibbling and grazing. Low ADPN in BED may also be associated with abnormal ghrelin levels, leading to an overactive hunger response—driving binge episodes (Fricke & Voderholzer, 2023). Individuals with BED are at increased risk of metabolic syndrome (Zhou et al., 2022), a cluster of conditions including obesity, high blood pressure, and insulin resistance. Low ADPN further exacerbates these conditions, creating a cycle of metabolic dysregulation that perpetuates binge eating behavior.

ADPN could also serve as a biomarker for metabolic health in individuals with BED (Table 2). Understanding the interplay between ADPN and the hormonal regulation of hunger, emotional eating, and reward pathways is crucial in developing effective treatments for this eating disorder (Campos et al., 2022).

### 5.2 Inflammatory bowel disease and irritable bowel syndrome

The gut–brain axis regulates bidirectional communication, influencing critical physiological processes. Dysregulation of this axis is implicated in IBD and IBS (Mayer et al., 2023). IBD is characterized by mitochondrial dysfunction, cytoskeletal disruptions, and metabolic dysregulation (Boldyreva et al., 2024). Alterations in adipokine levels, including ADPN, correlate with disease severity (Morshedzadeh et al., 2017). ADPN is reduced in IBD, particularly in Crohn's disease (CD) and ulcerative colitis (UC), conditions marked by chronic intestinal inflammation (Surdea‐Blaga et al., 2023).

IBS, which shares some pathophysiological features with IBD (Fairbrass et al., 2020), has also been associated with low ADPN levels, though findings remain inconsistent (Roczniak et al., 2022). ADPN exerts protective effects by suppressing pro‐inflammatory cytokines (TNF‐α, IL‐6, and IL‐1β) and promoting anti‐inflammatory IL‐10 production (Pan et al., 2023). Additionally, ADPN modulates immune responses by shifting macrophages from a pro‐inflammatory (M1) to an anti‐inflammatory (M2) phenotype, reducing intestinal inflammation. Intestinal barrier disruption is a hallmark of IBD, leading to increased permeability and bacterial translocation (Lechuga & Ivanov, 2017). ADPN enhances tight junction proteins, preserving barrier integrity and protecting epithelial cells from inflammatory damage (Cheru et al., 2019). However, its role remains complex, as some studies report elevated ADPN levels in IBD, possibly as a compensatory response to inflammation (Surdea‐Blaga et al., 2023). In CD, chronic inflammation drives intestinal fibrosis, leading to strictures and obstructions (Wang et al., 2022). Given its anti‐inflammatory and barrier‐protective roles, ADPN represents a potential therapeutic target. Moreover, ADPN may be implicated in the dysregulated ceramide signaling in the gut, favoring gut–brain axis dysregulation (Doll & Snider, 2024; Li et al., 2022). Enhancing ADPN signaling may mitigate fibrosis, reduce inflammation, and decrease the need for surgical intervention. Additionally, ADPN's correlation with symptom severity suggests its potential as a diagnostic and prognostic biomarker in IBS (Russo et al., 2018).

### 5.3 Obesity

ADPN plays a critical role in obesity‐related metabolic dysfunction (Han et al., 2022; Siqueira et al., 2023), with its levels inversely correlated with body fat, particularly visceral adiposity. Obesity is accompanied by a chronic inflammatory state and significantly reduced circulating ADPN (Kirichenko et al., 2022; Tural et al., 2023), which exacerbates insulin resistance, inflammation, and cardiovascular disease (CVD). ADPN enhances insulin sensitivity by activating the AMPK pathway, promoting glucose uptake and fatty acid oxidation. However, obesity‐driven overproduction of pro‐inflammatory cytokines, such as TNF‐α and IL‐6, suppresses ADPN, further amplifying inflammation and metabolic dysregulation (Siqueira et al., 2023).

Low ADPN levels are strongly associated with CVD risk due to impaired vascular function. ADPN exerts cardioprotective effects (Boydens et al., 2012) by reducing atherosclerotic plaque formation (Menzaghi & Trischitta, 2018), vascular inflammation, and endothelial dysfunction while regulating blood pressure. In obesity, these protective mechanisms are compromised, contributing to hypertension and increased cardiovascular risk.

Given ADPN's metabolic and anti‐inflammatory roles, therapeutic strategies aim to elevate its levels. Weight loss via diet and exercise has been shown to enhance ADPN concentrations, improving insulin sensitivity and reducing inflammation (Polito, Di Meo, et al., 2020; Polito, Monda, et al., 2020; Takao et al., 2021). Pharmacological interventions, such as thiazolidinediones (TZDs), also increase ADPN levels, offering potential metabolic benefits (Gastaldelli et al., 2021). Targeting ADPN pathways through lifestyle or pharmacological approaches may mitigate obesity‐related complications, highlighting its role in metabolic homeostasis and CVD prevention.

## 6 CONCLUSIONS

ADPN is emerging as an interesting molecule with potential implications as a therapeutic target and a biomarker in metabolic and inflammatory diseases, including eating disorders, IBD/IBS, and obesity.

Recent preclinical studies on murine models—although with the related limitations—have highlighted its effects on the stomach, which is an attractive therapeutic target for studying ADPN peripheral effects, this organ being a fundamental part of the complex network of the gut–brain axis. Therefore, its morpho‐functional alterations can induce dysfunction with repercussions at the central and systemic levels. ADPN's anti‐inflammatory and insulin‐sensitizing properties suggest its therapeutic relevance in conditions characterized by chronic inflammation and metabolic dysregulation. In eating disorders, ADPN's role in energy balance and inflammation modulation points out its potential in restoring metabolic homeostasis and reducing relapse risk. Similarly, its ability to regulate inflammation in IBD and obesity highlights its broader therapeutic implications. Additionally, ADPN may serve as a prognostic biomarker, with elevated levels in anorexia and reduced levels in obesity reflecting its role in energy regulation. In this scenario, given ADPN's multifaceted impact on metabolic and inflammatory pathways, understanding ADPN's mechanisms and targets is crucial for optimizing its application in personalized treatments for complex metabolic disorders (Baldelli et al., 2024; Choi et al., 2020; Robinson et al., 2011) to successfully embrace the variety of therapeutic interventions.

## FUNDING INFORMATION

No funding information provided.

## CONFLICT OF INTEREST STATEMENT

The authors declare no conflict of interest.

## ETHICS STATEMENT

Reviews are exempt from ethical approval at the host institution of the corresponding author.
